# Artificial intelligence in traditional medicine: evidence, barriers, and a research roadmap for personalized care

**DOI:** 10.3389/frai.2025.1659338

**Published:** 2025-09-09

**Authors:** Ketmanee Jongjiamdee, Pimnipa Pornwonglert, Nutnichar Na Bangchang, Pravit Akarasereenont

**Affiliations:** ^1^Center of Applied Thai Traditional Medicine, Faculty of Medicine Siriraj Hospital, Mahidol University, Bangkok, Thailand; ^2^Department of Pharmacology, Faculty of Medicine Siriraj Hospital, Mahidol University, Bangkok, Thailand; ^3^Siriraj Metabolomics and Phenomics Center, Faculty of Medicine Siriraj Hospital, Mahidol University, Bangkok, Thailand

**Keywords:** artificial intelligence, integrated medicine, personalized treatment, telemedicine, traditional medicine

## Abstract

**Background:**

Traditional medicine (TM) systems such as Ayurveda, Traditional Chinese Medicine (TCM), and Thai Traditional Medicine (TTM) are increasingly intersecting with artificial intelligence (AI).

**Objective:**

To synthesize how AI is currently applied to TM and to outline barriers and research needs for safe, equitable, and scalable adoption.

**Methods:**

We conducted a targeted narrative mini review of peer reviewed studies (2017–Aug 2025) retrieved from PubMed, Scopus, and Google Scholar using terms spanning TM (Ayurveda/TCM/TTM) and AI (machine learning (ML), natural language processing (NLP), computer vision, telemedicine. Inclusion favored studies with reported methods and, when available, performance metrics; commentary and preprints without data were excluded.

**Findings:**

Current evidence supports AI assisted diagnostic pattern recognition, personalization frameworks integrating multi source data, digital preservation of TM knowledge, telemedicine enablement, and AI supported herbal pharmacology and safety assessment. Reported performance varies and is context dependent, with limited prospective external validation.

**Limitations:**

Evidence heterogeneity, small datasets, inconsistent ontologies across TM systems, and nascent regulatory pathways constrain real world deployment.

**Conclusion:**

AI can augment TM education, research, and clinical services, but progress requires standards, culturally informed datasets, prospective trials, and clear governance. We propose a research roadmap to guide rigorous and ethical integration.

## 1 Introduction

**Figure 1:**
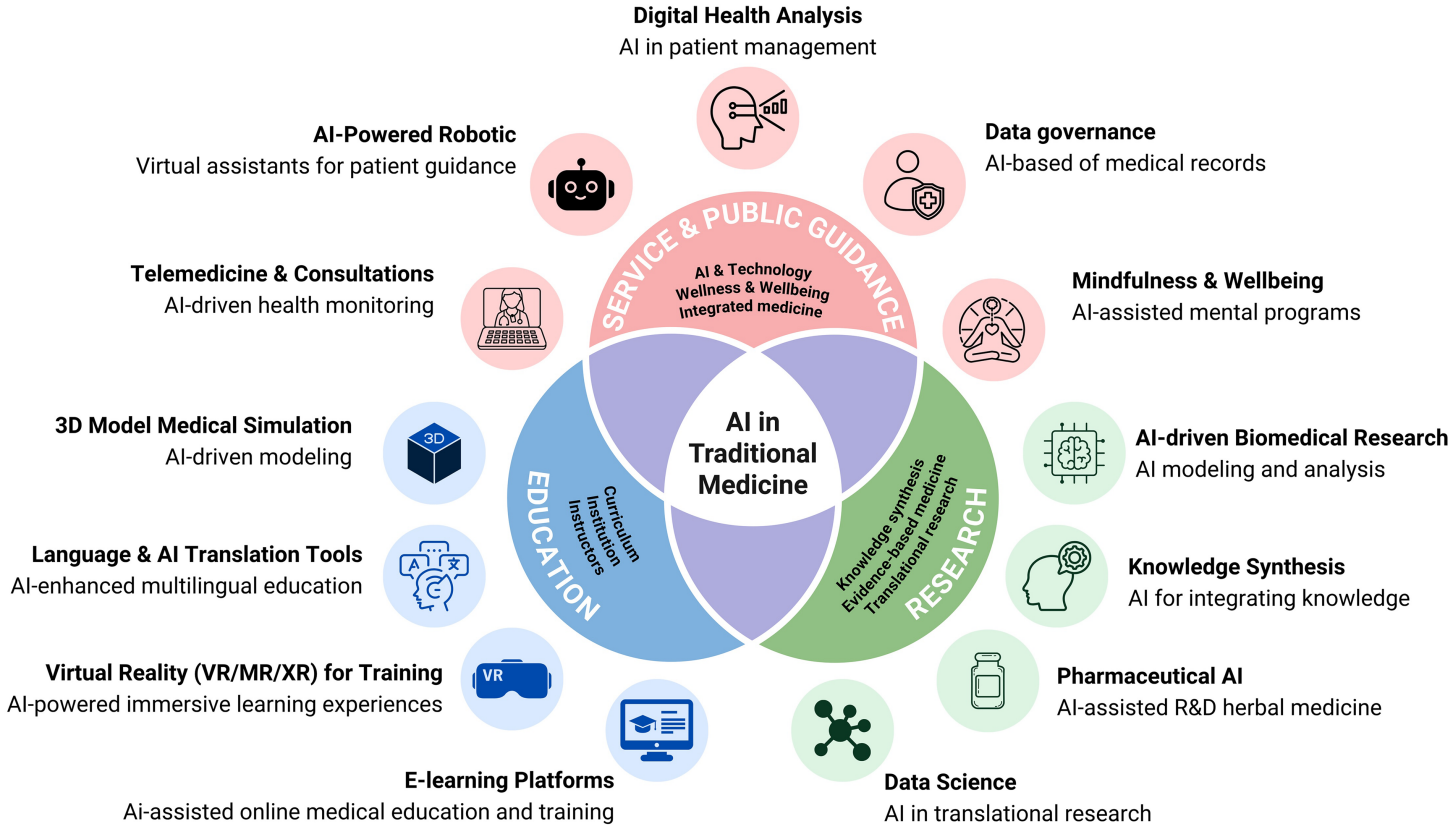
Applications of AI in traditional medicine across education, research, clinical service, and public guidance—mapping typical data flows (from acquisition to decision support) and example use cases (telemedicine, digital health analysis, AI assisted research).

The realm of traditional medicine (TM), encompassing practices such as Ayurveda, traditional Chinese medicine (TCM), Thai traditional medicine (TTM), and indigenous healing systems, holds a vast wealth of knowledge developed over centuries. These practices have successfully treated generations of people using natural methods rooted in holistic understanding and observing complex patterns in health and disease. However, the modern world presents new challenges: increasing demand for evidence-based practices, personalization of healthcare, and scalable, accessible treatment solutions. Artificial Intelligence (AI) stands at the intersection of these demands, offering transformative potential to elevate and expand the application of TM. AI’s capacity to process and analyze vast amounts of data, recognize complex patterns, and make predictive analyzes can revolutionize TM in multiple dimensions. This essay explores the potential benefits of integrating AI with TM, highlighting key areas where this synergy could bring significant advancements (Figure 1, Supplementary Figure S1).

## 2 Methods

### 2.1 Design and scope

We conducted a narrative mini review to map applications of AI across TM in diagnosis, personalized treatment, pharmacology, knowledge preservation, telemedicine and harmonization with modern care.

### 2.2 Search strategy

We searched PubMed/MEDLINE, Scopus and Google Scholar from inception through 15 Aug 2025 using combinations of: “Ayurveda,” “traditional Chinese medicine,” “Thai traditional medicine,” “traditional medicine,” AND “artificial intelligence,” “machine learning,” “deep learning,” “neural network,” “telemedicine,” “network pharmacology,” “tongue diagnosis,” “pulse diagnosis.”

### 2.3 Eligibility criteria

Included items were primary studies, reviews or practice papers that (i) applied AI/machine learning (ML) to TM relevant tasks; or (ii) discussed frameworks/validation relevant to AI in TM. We excluded items unrelated to AI, non-scholarly pieces, or anecdotal single case reports without analytical methods.

### 2.4 Selection and data extraction

Titles/abstracts were screened, with full text reviews for potentially eligible items. We extracted task, data type, TM system, AI method, validation approach and key performance indicators (e.g., accuracy, sensitivity, specificity, area under curve—AUC) when reported. Disagreements were resolved by discussion.

### 2.5 Synthesis

We conducted a narrative synthesis grouped by application domain, then provided a cross system comparative analysis (Ayurveda, TCM, TTM).

### 2.6 Limitations of the review

As a narrative mini review, we did not perform meta-analysis; heterogeneity of methods and reporting limited quantitative aggregation. Potential publication bias and language bias are acknowledged.

## 3 Enhanced diagnosis through AI pattern recognition

**Figure 2:**
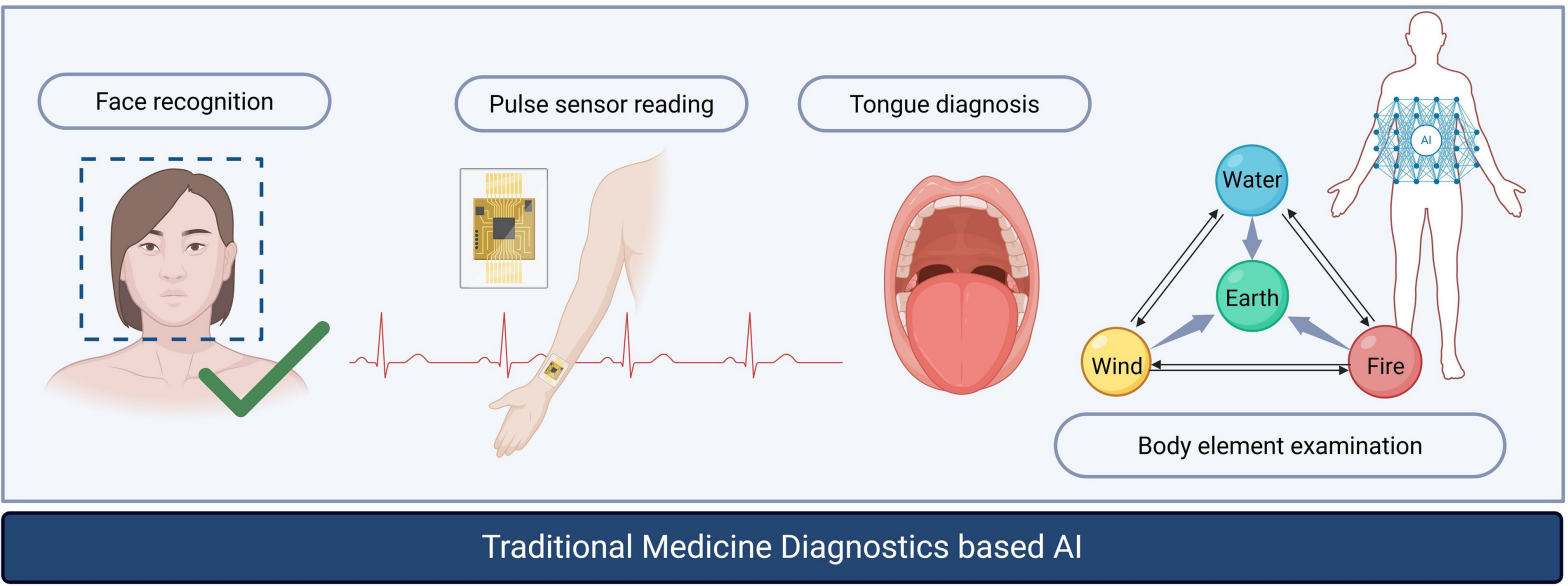
AI augmented diagnostic workflow in traditional medicine: face analysis, pulse sensor acquisition, tongue image analysis, and body element examination, illustrating points where models and standards are needed for reproducibility.

TM diagnostics—pulse reading, tongue inspection, body element examination, facial observation (Figure 2)—are rich pattern-recognition tasks but susceptible to inter-observer variability. Current evidence shows that ML has been used to differentiate TCM diagnostic patterns on curated datasets (e.g., dampness-heat in type 2 diabetes) and to structure acquisition protocols for sensor-based pulse assessment, with performance dependent on feature standardization and context (Liu et al., 2023; Tian et al., 2024; Zhou et al., 2024). Future potential includes interoperable signal standards, ontology alignment across systems, and external validation with multi-site cohorts (Lu et al., 2024).

### 3.1 Mini-case

AI-assisted pulse diagnosis: Prototype systems combine controlled pulse-wave acquisition with classifiers to support pattern interpretation. Early reports suggest improved reproducibility under standardized protocols; however, cross-device calibration, label consensus, and multi-site generalizability remain open challenges requiring prospective validation.

### 3.2 Comparative view

Comparative applications with representative, cited examples for each system. Ayurveda brings AI applications have been investigated for prakriti classification, diagnostic decision support, and remote triage. Narrative summaries and practice reports delineate workflows and data requirements (Ranade, 2024; Rastogi et al., 2022). TCM: ML aids in syndrome differentiation through inspection, inquiry, auscultation, olfaction, and palpation; cheminformatics and network pharmacology are vibrant fields of study (Liu et al., 2023; Tian et al., 2024; Zhou et al., 2024). TTM: Instruments for current body element evaluation and pulse assessment have surfaced, laying the groundwork for standardized data collection (Mahajaroensiri et al., 2017; Tantiwongsekunakorn et al., 2025). TM systems such as Ayurveda, TCM, and TTM are increasingly incorporating AI to create personalized treatment plans and diagnostic strategies for individual patients. In Ayurveda, strategies encompass rule-based and data-driven approaches for assessing body constitution (prakriti) and selection, facilitating tele-triage and remote assistance. In TCM, pattern-based approaches enhanced by machine learning surpass traditional methods in syndrome differentiation, further supported by technologies like tongue image analysis. TTM, on the other hand, utilizes body element profiling, incorporating device-assisted pulse signals and standardized examinations of body elements to guide personalized recommendations (See in Supplementary Table S1).

## 4 Personalized treatment plans with AI

A hallmark of TM is its focus on individualization—treatments tailored to a person’s specific constitution, lifestyle, and symptoms. Unlike many modern medical treatments, which often follow a one-size-fits-all approach, TM considers a person’s holistic profile. AI, with its predictive modelling and data analysis capabilities, can elevate this individualized approach (Ranade, 2024). AI systems can analyse genetic data, environmental factors, and health records to recommend highly personalized treatment plans (Lu et al., 2024). For example, in TCM, a practitioner might assess the balance of Yin and Yang or diagnose a condition based on pulse and tongue readings. Another, in TTM, a practitioner used the balance of body element concept to diagnose through present body element examination (Mahajaroensiri et al., 2017) and pulse reading. AI could augment this process by analyzing subtle biometric data that may be too complex for humans to process quickly, offering more precise and personalized diagnostic results (Liu et al., 2023; Tian et al., 2024). This combination of ancient practice and modern technology could lead to highly customized treatments that are effective and harmonious with an individual’s unique biology (Supplementary Figure S2).

## 5 Predictive analytics for treatment outcomes

TM often follows a holistic approach, treating the symptoms and underlying causes of illness. However, predicting the outcomes of specific treatments, especially in complex cases, can be challenging. AI’s predictive analytics tools can model potential outcomes based on patient data, offering a more data-driven approach to traditional treatments (Bae et al., 2024). By processing complex data and identifying intricate patterns, these intelligent systems can predict potential treatment candidates, understand disease pathology and verify target proteins with remarkable accuracy. For instance, researchers have developed sophisticated AI-driven models, such as multi-target-based polypharmacology prediction systems, that showcase the ability of AI to screen TCM compounds efficiently (Liu et al., 2021). This predictive capability would enhance both the safety and effectiveness of treatments, giving practitioners the confidence that their chosen therapies are optimal for each patient. Furthermore, it could help refine the criteria for patient selection, ensuring that traditional treatments are applied where they are most likely to have a positive impact (Bae et al., 2024).

## 6 AI in herbal pharmacology

AI enables large-scale curation of herbal knowledge, prioritization of bioactive compounds, target prediction, and interaction risk assessment (Zhou et al., 2024; Song et al., 2024). Importantly, safety models predicting risks such as drug-induced liver injury (DILI) based on chemical structure illustrate how AI can augment risk assessment for herb and herb–drug combinations (Liu et al., 2021). Future potential requires TM-specific external validation datasets and integration with pharmacovigilance.

## 7 Preservation and digitization of traditional knowledge

AI-assisted digitization makes scattered textual and oral knowledge searchable and interoperable, supporting education and research (Song et al., 2024; Srivastava and Upadhyay, 2024; Li, 2024). Standardized terminologies and cross-tradition mappings (e.g., TTM body elements, TCM patterns, Ayurvedic doshas) are prerequisites for multilingual corpora and knowledge graphs; expert-elicited resources on pulse characteristics by innate body elements in TTM provide anchors for such work (Tantiwongsekunakorn et al., 2025).

## 8 AI and telemedicine: expanding access to TM

AI-enabled teleconsultation can extend access to TM practitioners and support remote triage and monitoring (Rastogi et al., 2022). Current evidence is promising for feasibility and reach; future potential hinges on rigorous evaluation of diagnostic concordance, safety, equity, and patient understanding. See validation and reporting checklist for AI in TM (Supplementary Table S2).

## 9 Harmonizing traditional and modern medical approaches

AI can help integrate TM with biomedicine by identifying potential synergies and risks, including herb–drug interactions, promoting safer combined regimens (Maria, 2024; Alowais et al., 2023).

### 9.1 Limitations, risks and barriers to adoption

The integration of AI into TM faces significant hurdles that must be addressed for widespread adoption. A primary challenge lies in data quality and standardization, as the heterogeneous nature of data acquisition—from pulse sensors to tongue images—and variable annotations make it difficult to build generalizable models. To mitigate this, establishing shared protocols, calibration standards, and robust data governance is crucial. Additionally, bias and cultural context pose a substantial risk, as models trained on skewed demographics or single-center data may not be applicable across diverse populations. This can be countered by using multi-site, multi-ethnic datasets and conducting regular fairness audits. The interpretability and clinical acceptance of AI models are also major barriers; clinicians are often reluctant to trust “black-box” models. Mitigation strategies include developing explainable AI models, implementing case-based reasoning, and creating tools that keep clinicians actively involved in the decision-making loop. Furthermore, the lack of clear regulation and safety guidelines for AI in a clinical setting hinders its use, necessitating the development of clear risk-management plans and post-deployment surveillance. Protecting privacy and security is another critical concern, given the sensitive nature of digitized TM knowledge and patient data. This requires the use of privacy-preserving learning techniques, effective consent management, and secure infrastructure. Finally, infrastructure and cost constraints, particularly in resource-limited settings, can limit deployment. This can be addressed through the development of low-cost sensors, the use of edge AI for on-device processing, and a phased roll-out strategy.

### 9.2 Ethical and regulatory considerations

Principles include transparent model reporting, culturally respectful data practices and benefit-sharing, privacy-preserving analytics, pre-deployment risk assessment, post-deployment safety/drift monitoring, and alignment with regional regulations. In integrated care, AI should “augment—not replace -” practitioner judgment, with clear accountability and mitigation of automation bias.

### 9.3 Future research roadmap

Responsible adoption of AI in TM hinges on: (i) standardizing data capture; (ii) transparently reporting validation—including cross-validation and external test-set results where feasible; (iii) ensuring fairness, safety, and privacy; and (iv) co-design with practitioners and patients. These steps can help align TM’s strengths with modern evidence standards, improving access, quality, and safety of care.

## 10 Conclusion

The integration of AI into TM has the potential to bring about a new era of healthcare. By leveraging AI’s ability to process complex data, recognize patterns, and personalize treatments, TM can evolve to meet modern healthcare demands while preserving its rich heritage. This synergy between ancient wisdom and modern innovation holds the promise of more effective, accessible, and precise healthcare, benefitting individuals and societies worldwide. As AI technology continues to advance, its role in TM will likely grow, offering exciting possibilities for the future of holistic health and wellness.
