# Supplementary material for: Artificial intelligence in traditional medicine: evidence, barriers, and a research roadmap for personalized care
Source: Front Artif Intell. 2025 Sep 9;8:1659338. doi: 10.3389/frai.2025.1659338 (PMC12455356; doi:10.3389/frai.2025.1659338)
Supplement: Supplementary file 1 [file Data_Sheet_1.pdf]

Supplementary Material

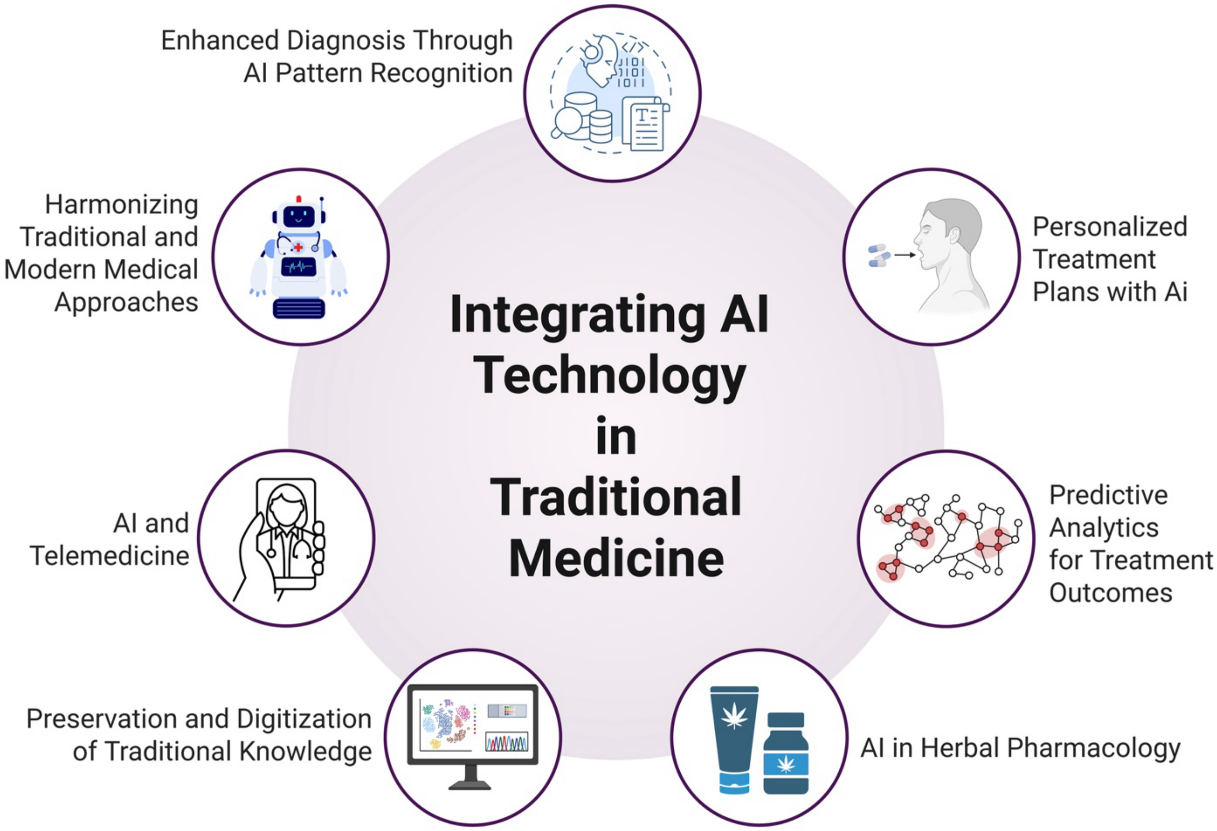

Figure S1. Conceptual framework linking six domains (diagnostics, personalization, herbal pharmacology, digitization, telemedicine, and harmonization) to AI capabilities.

Table S1. Comparative snapshot of AI for diagnostics/personalization across TM systems

Domain	Ayurveda	TCM	TTM	Shared gaps
**Diagnostic support**	Exploratory AI for Nadi Pariksha and tele triage	- ML classification of patterns<br><br>- Tongue image analysis	- Device aided pulse<br><br>- Body element assessment	- Small datasets<br><br>- Label/ontology mismatch<br><br>- Cross-device calibration<br><br>- External validation
**Personalization**	Rule based and data driven selection	Pattern based models with ML augmentation	Body element–based profiling and signals	- Transparent criteria<br><br>- Prospective outcome evaluation

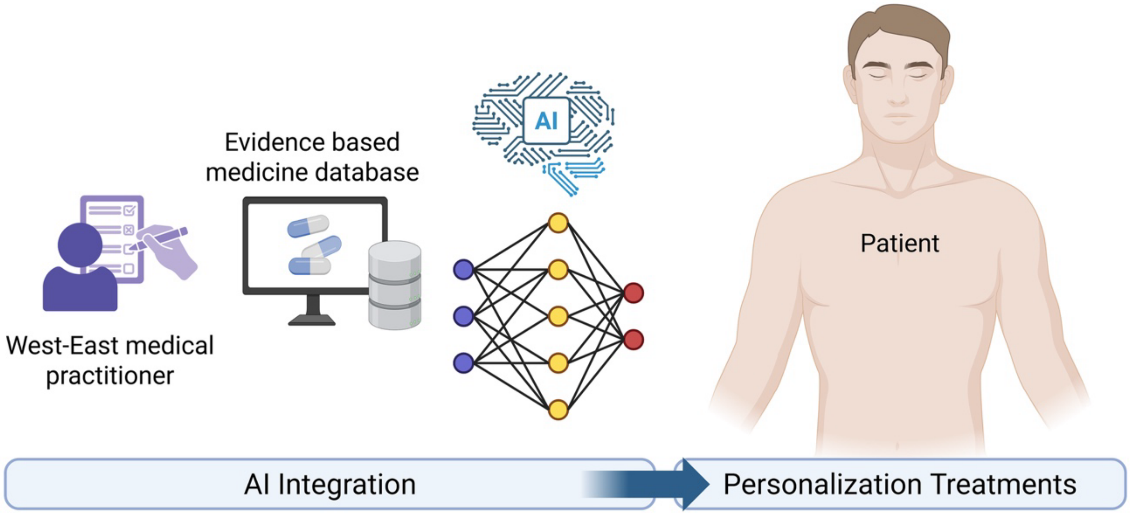

Figure S2. Personalization pipeline integrating practitioner inputs, patient data, and evidence databases via AI to inform individualized therapies.

Table S2. Validation and reporting checklist for AI in TM

	Validation &	Reporting Item
1	**Data provenance & cohort description**	Specify data sources (sites, timeframe, instruments/sensors), inclusion/exclusion, demographics (age/sex/ethnicity), language, and TM system (TTM/TCM/Ayurveda).
2	**Annotation protocol & reliability**	Describe labeling criteria, annotator expertise/training, and inter-rater reliability (e.g., semha, water, kappa).
3	**Data preprocessing & feature handling**	Report signal/image/text preprocessing (resolution, filtering, normalization, de-identification) and feature engineering.
4	**Model specification**	State model family/version, pipeline, key hyperparameters, and training setup (software, hardware, random seeds).
5	**Split strategy & validation design**	Define train/validation/test splits; use k-fold or nested CV as appropriate; report any external test set (site/time split) and anti-leakage measures.
6	**Class balance, sample size & missing data**	Provide class prevalence, sample size justification, and handling of missing data (imputation/exclusion).
7	**Primary outcomes & metrics**	Pre-specify primary metric(s) and thresholds. Classifiers: accuracy, F1, sensitivity/specificity, ROC-AUC (and PR-AUC for imbalance), confusion matrix, calibration. Regressions: MAE/RMSE and calibration.
8	**Uncertainty & statistical testing**	Report confidence intervals (e.g., bootstrap), significance tests, and sensitivity analyses.
9	**Fairness & subgroup performance**	Evaluate performance across subgroups (sex/age/ethnicity/site/device) and note mitigation for bias.
10	**Robustness & ablation**	Provide robustness checks (noise, perturbations, out-of-distribution) and ablation studies for key components.
11	**Clinical utility & human factors**	Describe intended use, decision thresholds, workflow integration, time to test result (TTR), and decision curve analysis (DCA) or clinical endpoints.
12	**Safety, privacy & governance**	Outline risk management, human-in-the-loop oversight (HITL), privacy/security measures, and regulatory context.
13	**Reproducibility & transparency**	Provide data/code availability statements where possible, versioning, and adherence to domain-appropriate AI reporting guidance.
